# Evaluation of Factors Affecting Fluoride Release from Dental Sealants: A Systematic Review

**DOI:** 10.3390/ma18235350

**Published:** 2025-11-27

**Authors:** Maciej Dobrzyński, Sylwia Klimas, Agnieszka Kotela, Zuzanna Majchrzak, Julia Kensy, Marzena Laszczyńska, Witold Świenc, Natalia Grychowska-Gąsior, Magdalena Fast, Jacek Matys

**Affiliations:** 1 Department of Pediatric Dentistry and Preclinical Dentistry, Wroclaw Medical University, Krakowska 26, 50-425 Wroclaw, Poland; sylwia.klimas@student.umw.edu.pl (S.K.); julia.kensy@student.umw.edu.pl (J.K.); 2 Medical Center of Innovation, Wroclaw Medical University, Krakowska 26, 50-425 Wroclaw, Poland; kotela.agnieszka@gmail.com (A.K.); zuzanna.h.nawrocka@gmail.com (Z.M.);; 3 Dental Surgery Department, Wroclaw Medical University, Krakowska 26, 50-425 Wroclaw, Poland; witold.swienc@umw.edu.pl; 4 Department of Dental Prosthetics, Wroclaw Medical University, Krakowska 26, 50-425 Wroclaw, Poland; natgrychowska@gmail.com; 5 Department of Drug Form Technology, Wroclaw Medical University, Borowska 211 A, 50-556 Wroclaw, Poland; magdalena.fast@umw.edu.pl

**Keywords:** antibacterial activity, dental sealants, fluoride release, glass ionomer cement, remineralization

## Abstract

Introduction: Fluoride-releasing sealants play a crucial role in caries prevention by providing both mechanical protection and controlled ion release. However, the rate and duration of fluoride emission vary depending on material composition and environmental conditions. Objectives: This systematic review aimed to identify and analyze factors influencing fluoride release and recharge capacity from contemporary dental sealants. Methods: A comprehensive literature search was conducted in September 2025 across PubMed, Scopus, Web of Science, Embase, and WorldCat using the keywords “fluoride release” AND “sealant.” Study selection followed PRISMA 2020 guidelines and predefined PICO-based eligibility criteria. Out of the 375 records initially identified, 16 studies fulfilled the inclusion criteria and were incorporated into the qualitative analysis. Results: Glass-ionomer and resin-modified glass-ionomer sealants (e.g., Fuji VII, Fuji IX, Fuji II LC, Ketac Molar, Vitremer) consistently demonstrated higher fluoride release and superior recharge capacity compared with resin-based sealants (e.g., Clinpro, Helioseal F, BeautiSealant). Most studies reported a biphasic fluoride-release pattern, characterized by an initial burst within the first 24–48 h followed by a slower, sustained phase lasting several weeks. Environmental parameters, including storage medium, pH, and temperature, significantly affected fluoride-release kinetics. Additional outcomes such as enamel remineralization, antibacterial activity, microleakage, and surface hardness were also reported. Conclusions: Both material composition and environmental factors substantially influence fluoride release from dental sealants. Glass-ionomer–based materials demonstrate more durable and higher fluoride release, with acidic conditions enhancing ion diffusion. Clinically, these materials are particularly beneficial for patients at high caries risk, whereas resin-based sealants remain preferable in low-risk cases due to better retention and esthetics.

## 1. Introduction

Dental caries is among the most common chronic diseases, particularly affecting the occlusal surfaces of molars, where deep pits and fissures facilitate bacterial accumulation and plaque retention [1,2,3,4]. Although fluoride provides effective protection on smooth enamel surfaces, its preventive efficacy is limited in these complex anatomical areas [5,6,7,8,9,10]. Pit and fissure sealants serve as an essential adjunct in caries prevention by forming a thin physical barrier that isolates enamel from the oral environment, thereby reducing bacterial colonization and the risk of decay formation [11,12,13]. This protective layer not only prevents the development of new lesions but can also arrest the progression of early non-cavitated caries [14,15,16]. Clinical research indicates that molars sealed soon after eruption remain caries-free for significantly longer periods compared with unsealed teeth [17]. Moreover, modern sealant materials often incorporate fluoride-releasing components that enhance remineralization and exhibit antibacterial effects [18].

Beyond their general preventive value, fluoride-releasing sealants play a particularly important role in specific clinical contexts. In pediatric patients, newly erupted molars with deep and narrow fissures are particularly prone to plaque accumulation and are often difficult to isolate adequately [17,18,19]. Under such conditions, the moisture tolerance and chemical adhesion of glass ionomer–based sealants make them advantageous, providing reliable protection even when complete isolation cannot be achieved [18]. Similarly, in individuals with elevated caries risk, such as those with orthodontic appliances or limited ability to maintain proper oral hygiene due to impaired motor function, fluoride-releasing materials offer an additional benefit by promoting remineralization and inhibiting bacterial activity at the enamel-sealant interface [20]. Consequently, the dual action of mechanical sealing and controlled fluoride release makes these materials a cornerstone of comprehensive, patient-tailored caries prevention strategies in both children and adults [19].

Fluoride release from pit and fissure sealants depends on several interrelated factors, including material composition, environmental conditions, and fluoride recharge potential. The long-term anticariogenic properties of glass ionomer and resin-modified glass ionomer cements stem from the ion exchange process between the glass particles and the polymeric acid matrix, leading to sustained fluoride emission [21,22]. However, their mechanical strength is lower than that of resin-based sealants. GICs chemically bond to enamel and are tolerant to moisture, which is particularly advantageous when isolation is difficult, especially in pediatric dentistry [18,23]. The oral environment, particularly fluctuations in pH and exposure to topical fluoride agents, influences both the rate and duration of ion release. Notably, many GIC-based materials can be “recharged,” meaning they absorb fluoride from toothpaste or professional applications and subsequently release it over time [17]. Resin-based sealants and compomers exhibit lower fluoride release; however, they compensate for this limitation with superior retention and wear resistance [18,24,25,26]. Therefore, the long-term clinical effectiveness of pit and fissure sealants depends on the balance between mechanical durability, fluoride release capacity, and the potential for recharge under oral conditions [17,27,28].

**Figure 1:**
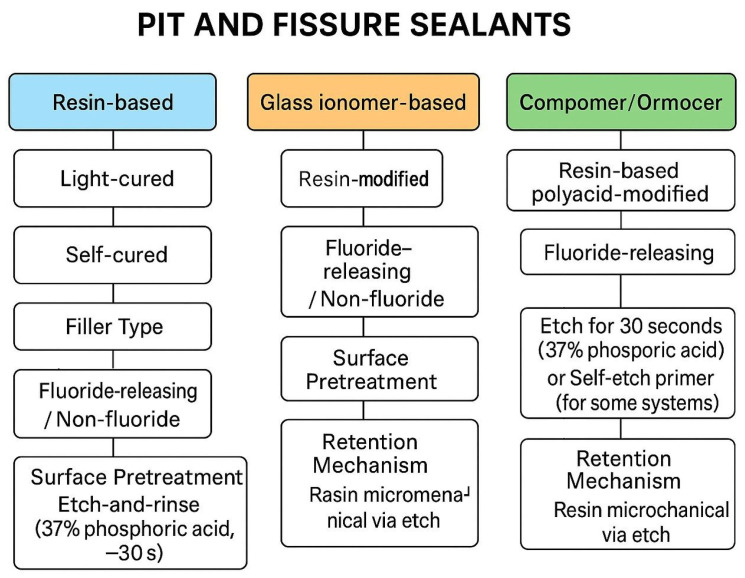
Classification of Pit and Fissure Sealants Based on Material Type and Retention Mechanism.

The main categories of dental sealants include glass ionomer cements (GICs), resin-based sealants, and hybrid sealants such as compomers and giomers [29]. GICs set through an acid–base reaction between fluoroaluminosilicate glass and polyalkenoic acid, forming ionically cross-linked matrices that enable fluoride ion transport via diffusion and ion exchange [21,30,31,32]. These materials are hydrophilic and exhibit water sorption, which facilitates the continuous movement of ions [30]. GICs demonstrate an initial burst- a rapid initial liberation of fluoride ions, followed by a prolonged, low-level phase that persists over time [30,32]. They can also be recharged with fluoride after topical exposure and subsequently re-release it [30,33]. Resin-based sealants are cross-linked polymers with a predominantly hydrophobic composition, which limits ion diffusion through the material [31,34]. When present, the magnitude and duration of fluoride release from resin sealants are generally lower than those reported for GICs [34,35,36,37]. However, resin-based sealants are characterized by excellent handling properties, high wear resistance, and durable retention on enamel surfaces [34]. Hybrid sealants constitute a distinct group that includes compomers and giomers [29,38,39,40]. Compomers are resin-based materials containing ion-releasing glass particles that allow for limited fluoride release, typically lower than that of conventional glass ionomer cements [31,39,41]. Giomers consist of resin matrices incorporating surface pre-reacted glass ionomer (S-PRG) fillers, which act as micro-reservoirs for multiple ions, including fluoride [38,39]. These S-PRG fillers gradually release fluoride into the surrounding environment while preserving the handling and mechanical characteristics of resin composites [38,39]. Moreover, giomer and other S-PRG–based materials can be recharged with fluoride following exposure to topical fluoride agents [38]. A detailed classification of these fissure sealants is presented on Figure 1.

Although fluoride release from dental materials contributes to caries prevention, the amounts entering the oral cavity must remain within safe physiological limits. Fluoride is released only in trace quantities, far below levels associated with biological harm. Toxicological data indicate that early symptoms of fluoride overexposure appear at approximately 1 mg F^−^/kg body weight, while the probably toxic dose is around 5 mg F^−^/kg and about 8 mg F^−^/kg is considered safely tolerated. Much higher exposures (14–28 mg F^−^/kg) may be potentially lethal, with a certain lethal dose estimated at 32–64 mg F^−^/kg for adults and about 15 mg F^−^/kg for children [11,42,43,44,45]. These thresholds are several orders of magnitude higher than the amounts released from resin-based materials, which are therefore generally regarded as safe for clinical use.

This systematic review aims to comprehensively evaluate the influence of both material composition and environmental conditions on the release and recharge capacity of fluoride from contemporary dental sealants. By systematically analyzing available evidence, this study seeks to identify key factors determining the rate, duration, and pattern of fluoride release, as well as their implications for the clinical performance and preventive effectiveness of various sealant types.

## 2. Materials and Methods

### 2.1. Focused Question

In dental sealants applied to teeth (P), how do variations in formulation—including differences in fluoride content, material type, and incorporation of bioactive additives (I)—compared with conventional non-fluoride sealants or untreated tooth surfaces (C)—influence the amount and pattern of fluoride release over time (O)?

### 2.2. Protocol

**Figure 2:**
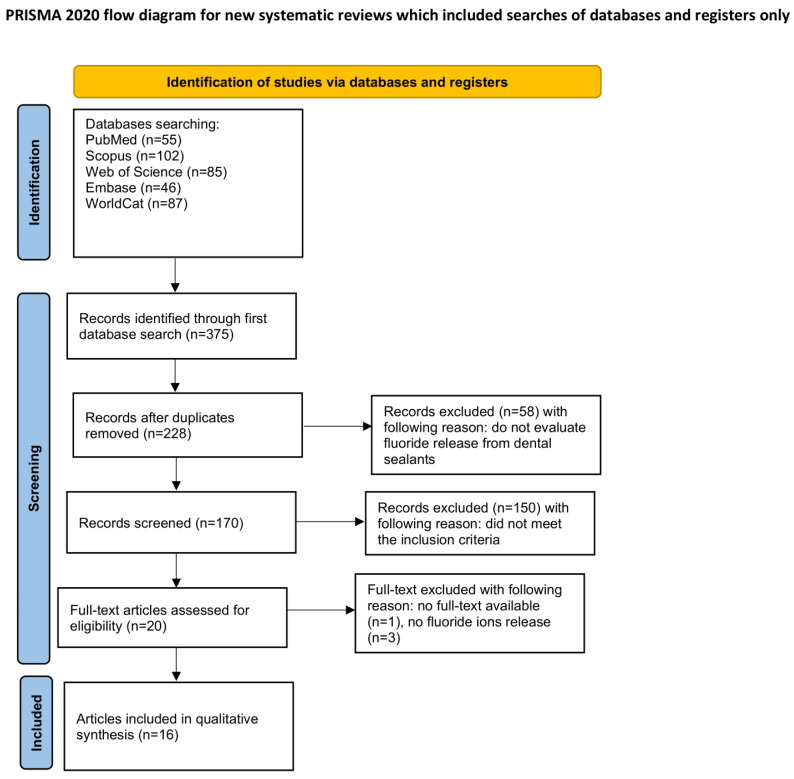
The PRISMA 2020 flow diagram.

The procedure for identifying and selecting studies followed the PRISMA framework for systematic reviews, as presented on Figure 2. The corresponding review protocol was prospectively registered in the Open Science Framework (OSF) database under the link: https://osf.io/pxc9h/ (accessed on 7 October 2025).

### 2.3. Eligibility Criteria

The following criteria were applied to determine study eligibility for inclusion in this review [46,47,48,49,50]:

- Studied the fluoride release behavior of dental sealant materials.
- Presented original experimental data.
- Conducted as either in vitro or in vivo investigations.
- Performed on extracted or natural teeth.
- Published in English.
- Available in full-text format.

The exclusion criteria were defined as follows [46,47,48,49,50]:

- Studies assessing outcomes unrelated to fluoride release.
- Research conducted on artificial or synthetic specimens.
- Publications in languages other than English.
- Articles published before the year 2000.
- Systematic reviews, narrative reviews, or clinical reports.
- Editorials, commentaries, or letters to the editor.
- Papers for which the full text was not accessible.
- Duplicate entries identified during screening.

### 2.4. Information Sources, Search Strategy, and Study Selection

A comprehensive literature search was performed up to September 2025 using the PubMed, Scopus, Web of Science (WoS), Embase, and WorldCat databases. The search aimed to identify studies that met the predefined eligibility criteria and investigated factors influencing fluoride release from dental sealants. The primary search terms were “fluoride release” and “sealant”, combined using the Boolean operator AND. Search syntax was adapted to the requirements of each database. Only studies with an accessible full-text version were considered for inclusion.

The specific search strategies for each database were as follows:

- PubMed: “fluoride release” [All Fields] AND “sealant” [All Fields].
- Scopus: TITLE-ABS-KEY (“fluoride release” AND “sealant”).
- Web of Science: TS = (“fluoride release” AND “sealant”).
- Embase: (‘fluoride release’:ti,ab AND ‘sealant’:ti,ab).
- WorldCat: “fluoride release” AND “sealant”.

Only studies with an accessible full-text version were considered for inclusion. Non-English studies were excluded due to limited access to accurate translation and the potential risk of misinterpretation. Similarly, studies that did not involve natural specimens were expelled to ensure clinical relevance and methodological consistency across included papers. Other reviews, clinical reports or editorials were excluded to avoid the risk of lacking primary data suitable synthesis.

### 2.5. Data Collection Process and, Data Items

Six independent reviewers (S.K., J.K., A.K., M.L., Z.N., and W.Ś.) participated in the screening and selection of studies according to the predefined inclusion criteria. Key details were extracted from each eligible study, including information on the first author, publication year, study type, article title, fluoride release findings, and the dental sealant tested. All collected data were organized and tabulated using a standardized Microsoft Excel 365 form (Microsoft Excel 365, Version 2505, Build 16.0.18827.20102, 64-bit).

### 2.6. Risk of Bias and Quality Assessment

In the initial screening phase, all reviewers independently evaluated the titles and abstracts of the identified studies to minimize potential selection bias. Inter-reviewer agreement was quantified using Cohen’s kappa coefficient. Any discrepancies concerning study inclusion or exclusion were resolved through group discussion until a unanimous decision was achieved.

### 2.7. Quality Assessment

Two blinded assessors (J.M. and M.D.) independently appraised the methodological rigor of the included studies using the JBI Critical Appraisal Checklist designed for quasi-experimental (non-randomized) designs [51]. This tool was selected because it corresponds to the study designs under review and provides a well-established, transparent, and reproducible framework for assessing study quality in line with international methodological standards. The checklist consists of nine core items addressing issues such as: the clarity of the cause–effect relationship, comparability between groups, similarity of treatment conditions apart from the intervention, presence of a control group, frequency and timing of outcome measurements, adequacy of follow-up, reliability of outcome assessment, and appropriateness of statistical methods. Each criterion was rated using one of four possible responses: yes, no, unclear, or not applicable. Disagreements between the two reviewers were resolved through discussion until consensus was reached. Inter-rater reliability was analyzed using Cohen’s kappa statistic, calculated with MedCalc software (version 23.1.7; MedCalc Software Ltd., Ostend, Belgium). The resulting kappa coefficient of 0.88 indicated excellent agreement and high consistency between reviewers.

## 3. Results

### 3.1. Study Selection

An extensive database search encompassing PubMed, Scopus, Web of Science, Embase, and WorldCat identified 375 potentially pertinent studies. After eliminating 147 duplicates, the remaining titles and abstracts were screened, and studies that did not evaluate the in vitro performance of dental sealants were excluded. Subsequently, 20 articles were retrieved for full-text review. Among these, three studies were excluded for not fulfilling the eligibility criteria [52,53,54], while one article was removed due to inaccessible full text [55]. Ultimately, 16 studies met all inclusion criteria and were included in the qualitative synthesis [56,57,58,59,60,61,62,63,64,65,66,67,68,69,70,71]. A meta-analysis was not performed due to substantial methodological heterogeneity across the included studies—particularly in study design, storage media, measurement intervals, and analytical techniques—which precluded meaningful quantitative pooling of fluoride-release outcomes.

### 3.2. General Characteristics of the Included Studies

A total of sixteen studies met the inclusion criteria for this systematic review, comprising mainly in vitro investigation [56,57,58,59,60,61,62,63,65,66,67,68,70,71] and two in vivo studies [64,69]. These studies evaluated a wide range of fluoride-releasing sealants and restorative materials under diverse experimental conditions. Most of the included papers investigated glass-ionomer and resin-modified glass-ionomer sealants such as Fuji VII, Fuji IX, Fuji III, Fuji III LC, Fuji II LC, Ketac Molar, ProSeal, and Vitremer [56,59,60,61,62,64,66,67,69,71], which consistently exhibited higher fluoride release and greater recharge potential compared with resin-based sealants including Clinpro, Helioseal F, BeautiSealant, Teethmate F-1, Conseal F, FluroShield, and Fissurit F [56,57,58,59,61,62,63,64,65,67,68,70].

Several studies also examined materials incorporating functional additives such as bioactive glass nanoparticles (NovaMin), surface pre-reacted glass-ionomer (S-PRG) fillers, or antibacterial monomers, which demonstrated enhanced ion release and antimicrobial activity [65,66]. Across most experiments, an initial “burst” of fluoride release occurred within the first 24 h, followed by a gradual decline to a steady-state phase [57,58,59,60,61,64,68,69]. Environmental factors including the immersion medium and pH fluctuations significantly affected fluoride-release kinetics [58,59,70,71].

Glass-ionomer-based materials generally showed superior fluoride recharge capacity following topical fluoride exposure and promoted increased enamel fluoride uptake and remineralization potential [56,58,61,62,65,66,71]. In addition to ion-release measurements, several studies also evaluated antibacterial effects [66], surface hardness [59,68,70,71], and microleakage [65,68]. Collectively, fluoride-releasing sealants demonstrated distinct advantages in preventing demineralization and enhancing the cariostatic efficacy of enamel.

The general characteristics of the included studies are summarized in Table 1.

### 3.3. Main Study Outcomes

#### 3.3.1. Sample Design

The included studies exhibited substantial variation in sample design, with differences in specimen type, geometry, and preparation methods. Most investigations employed standardized discs or blocks fabricated from the tested materials [57,58,59,60,61,62,63,65,66,67,68], while several studies used enamel substrates or whole teeth—extracted human or bovine premolars, molars, and incisors—to better replicate clinical conditions [56,67,70,71]. Two in vivo studies by Ananda et al. [64] and Kamala et al. [69] evaluated sealants directly in children’s oral environments. Sample sizes ranged from fewer than 10 specimens per group in small-scale laboratory studies [57,58,59,60,61,62,63,65,66,67,68] to over 50 samples in large clinical or experimental trials [64,69,71].

#### 3.3.2. Storage Conditions

There was notable heterogeneity in the immersion media and test environments used across the studies. Deionized or distilled water was the most common storage medium [57,58,60,61,62,63,65,66,67,68], typically maintained at 37 °C to simulate intraoral temperature and replaced daily or weekly. Some studies employed artificial saliva formulations with near-neutral pH [56,59], while others simulated cariogenic conditions using acidic or pH-cycling models with alternating demineralizing and remineralizing phases [59,70,71]. Specific ionic or acidic solutions, such as 0.9% NaCl [58] or citric acid [59], were also used to evaluate fluoride release under stress conditions. Immersion volumes ranged widely—from 0.5 mL per sample [56] to 10 mL [60,61]. In contrast, the two in vivo studies naturally used the oral cavity as the storage environment for fluoride diffusion [64,69].

#### 3.3.3. Measurement Methods and Timing

Fluoride quantification methods varied considerably. The fluoride ion-selective electrode was the primary analytical tool used in most studies [58,59,60,61,62,63,64,65,66,67,68,69,70], frequently in conjunction with TISAB III buffer to maintain constant ionic strength and pH [60,61,64,65,66,68,70]. Only one study, by Şişmanoğlu et al. [57], used spectrophotometric analysis. Measurement intervals also differed substantially: early-phase monitoring often involved hourly or daily sampling to capture the initial “burst” release [56,57,58,59,60,61,62,65,68,70], while longer-term studies extended up to several weeks or months with less frequent sampling [63,66,69]. Multiple studies included fluoride “recharge” protocols—immersing specimens in topical fluoride products (acidulated phosphate fluoride gel, 0.05% NaF, Profluorid Varnish, or MI Paste Plus)—to assess post-exposure release and recharging ability [61,62,63,65,67].

#### 3.3.4. Fluoride Release Results

Marked variability was observed in both the magnitude and duration of fluoride release, largely dependent on material composition and test conditions.

Ei et al. [56] reported peak fluoride release on day 2, with Fuji VII maintaining the highest cumulative values after 14 days, whereas Teethmate F-1 and Clinpro declined sharply after day 4. Şişmanoğlu et al. [57] recorded an initial BeautiSealant release of 5.33 ppm on day 1, tapering over 28 days, while Fissurit F exhibited slightly higher residual release. Fita et al. [58] and Kantovitz et al. [59] similarly described an early “burst effect” followed by stabilization by day 15. In acidic environments, Vitremer released the most fluoride initially, whereas Ketac Molar surpassed it in later phases, and FluoroShield consistently exhibited the lowest release.

Across studies, glass-ionomer and resin-modified glass-ionomer sealants consistently outperformed resin-based materials in both initial and sustained fluoride release. Fuji-based sealants showed particularly strong and durable release profiles [56,60,61,62,63,64,66,67,68,69,71]. Prapansilp et al. [60] demonstrated that Fuji VII released 8.27 ppm on day 1 when prepared according to manufacturer instructions, while Asvanund et al. [61] found that the same material achieved 6.62 ppm initially and 15.42 ppm after recharge with acidulated phosphate fluoride. Dionysopoulos et al. [62] reported that the cumulative 28-day release of the glass ionomer FX II (408.6 ± 45.7 µg/cm^2^) exceeded that of Teethmate F-1 (89.5 ± 12.3 µg/cm^2^) and BeautiSealant (33.3 ± 4.9 µg/cm^2^). Poggio et al. [63] found that Fuji Triage maintained a continuous increase in fluoride output up to 8.0 ppm by day 49, outperforming Fissurit FX and Grandio Seal, while fluoride varnish recharge raised release values dramatically. In vivo, Ananda et al. [64] and Kamala et al. [69] confirmed that Fuji IX GP, Fuji III, and Ketac Molar released significantly more fluoride into saliva and plaque than resin-based materials.

Resin-based sealants displayed substantially lower release levels. Fita et al. [58] and Fan et al. [65] observed that Clinpro and FluoroShield exhibited similar low cumulative release (~5 ppm after 14 days), and that the inclusion of bioactive glass (NovaMin) did not enhance fluoride output. Koga et al. [67] likewise found that Helioseal F released the least fluoride compared with Teethmate F-1 and glass-ionomer groups. Overall, most studies reported an initial 24 h release peak followed by a gradual decline to baseline levels, with glass-ionomer-based materials consistently exhibiting higher fluoride output, better recharge capacity, and stronger cariostatic potential.

#### 3.3.5. Additional Findings

Beyond fluoride-release performance, several studies evaluated complementary material properties. Ei et al. [56] demonstrated that Teethmate F-1 provided the most pronounced anti-demineralization effect among tested sealants. Prapansilp et al. [60] found that decreasing the powder-to-liquid ratio increased fluoride solubility and diffusion. Glass-ionomer materials generally combined the highest fluoride output with superior recharging behavior, making them more effective fluoride reservoirs [61]. Studies by Kaga et al. [66] showed that sealants containing S-PRG fillers released multiple ions (fluoride, strontium, and boron), contributing to antibacterial activity. Experimental sealants incorporating bioactive glass or antibacterial monomers exhibited improved sealing and lower microleakage compared to commercial products [65]. Kantovitz et al. [59] and Lobo et al. [70] reported that acidic environments decreased surface hardness and increased roughness, whereas some resin sealants such as FluoroShield showed greater resistance to degradation. Leão et al. [71] confirmed that both glass-ionomer and resin-modified glass-ionomer materials enhanced enamel hardness and fluoride uptake, further supporting their cariostatic advantages.

The detailed results of the included studies are demonstrated in Table 2.

### 3.4. Quality Assessment

**Figure 3:**
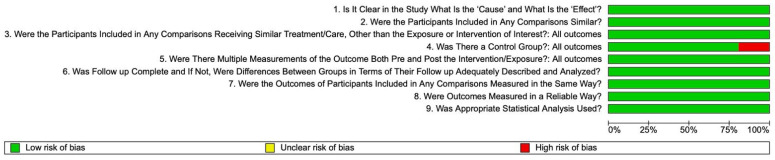
Risk of bias across the included studies.

Across all nine JBI appraisal items, thirteen studies achieved the maximum score of nine positive responses [56,60,61,62,63,64,65,66,67,68,69,70,71], while three studies obtained eight positive responses [57,58,59]. All sixteen studies included in this review were judged to have a low risk of bias, with overall quality scores ranging from 7 to 9. No study fell below the predefined threshold for low methodological risk, and therefore none were categorized as having a moderate or high risk of bias. To improve transparency and facilitate readers’ interpretation of methodological robustness, a risk-of-bias plot (Figure 3) has been incorporated to visually summarize the JBI scoring distribution, and detailed item-by-item assessments for each study are provided in Supplementary Table S1.

## 4. Discussion

The purpose of conducting this systematic review was to assess the fluoride release behavior, recharge potential, and corresponding physicochemical and biological features of current fluoride-releasing sealant materials. Among the sixteen studies included, comprising both in vitro and in vivo investigations, glass-ionomer and resin-modified glass-ionomer materials—such as Fuji VII, Fuji IX, and Ketac Molar—consistently demonstrated higher fluoride release and superior recharge capacity compared with resin-based sealants, including Clinpro, BeautiSealant, and Fissurit F. The analyzed data confirmed a characteristic fluoride-release pattern, marked by an initial “burst” within the first 24 h followed by a gradual stabilization phase, consistent with previous observations by Dionysopoulos et al. and Poggio et al. [62,63]. Environmental factors, such as pH, immersion medium, and material composition, were also shown to significantly influence fluoride kinetics, in agreement with the findings of Kantovitz et al. and Fita et al. [58,59]. Consistent with the studies by Asvanund et al. and Prapansilp et al. [60,61], glass-ionomer-based materials exhibited greater enamel fluoride uptake, higher remineralization potential, and a more sustained ion-exchange capacity. To provide greater interpretative depth, the present review further highlights mechanisms responsible for the superior recharge capacity of GICs and S-PRG-based materials. In GICs, the polyalkenoate matrix contains loosely cross-linked bridges that facilitate bidirectional ion diffusion, enabling both prolonged fluoride release and efficient re-uptake effect after exposure to external fluoride sources. In contrast, S-PRG fillers possess a multi-shell structure, formed in pre-reaction, which generates zones capable of repeated ion exchange with environment, which not only supports sustained fluoride availability, but also contributes to release of auxiliary ions, leading to buffering and antibacterial properties. These particular differences help to explain the consistently higher recharge behavior observed in these material categories compared with resin-based sealants, which due to theirs dense polymer network have ionic transport restricted. Overall, these findings highlight the clinical importance of fluoride-releasing sealants, particularly glass-ionomer-based formulations, which combine durable chemical bonding to enamel with long-term cariostatic activity, effectively contributing to the prevention of demineralization and secondary caries formation.

The present review confirms that the chemical composition and structural characteristics of sealant materials are primary determinants of fluoride-ion release behavior. Glass-ionomer-based sealants, including Fuji VII [56,60,61,69], Fuji IX GP [64,71], FX-II [62], Vitremer [59,70], Ketac Molar [59,64], Fuji Triage [63,67], Fuji III LC [66,67], Fuji III [67,69], and Fuji II L [71], consistently demonstrated the highest and most sustained fluoride release. This pattern can be attributed to the porous and hydrophilic structure of the glass-ionomer matrix, which facilitates continuous ion diffusion and allows for fluoride recharge following exposure to topical fluoride sources. Comparable results were reported by Wiegand et al. [72] and Ng et al. [29], who identified the ion-exchange capability of glass-ionomer systems as a critical factor supporting prolonged fluoride emission. In contrast, resin-based sealants—such as Clinpro Sealant [56,57,61,65,68,70], Concise [61,70], Teethmate F-1 [56,62,64,67], Helioseal F [57,58,64,67], Helioseal F Plus [58], Conseal F [58], Arkona [58], Fissurit F [57,62], Fissurit FX [63], FluroShield [59,65], Grandio [63,68], and Filtek Z250XT [71]—exhibited lower fluoride release and a shorter duration of ion availability than glass-ionomer or S-PRG–based materials, due to the limited permeability of their dense polymer networks. Hybrid sealants containing surface pre-reacted glass (S-PRG) fillers, such as BeautiSealant [57,62] and Beautifil II [71], showed intermediate but stable fluoride release. Kaga et al. [66] also demonstrated that S-PRG fillers can release additional ions, including strontium and boron, which may enhance antibacterial and buffering properties [29]. Environmental parameters, particularly pH fluctuations and the composition of the storage medium, were likewise shown to significantly influence fluoride-release kinetics [58,59,70]. The influence of pH on fluoride release has notable clinical implications, as acidic environments have been shown to accelerate fluoride diffusion from both glass-ionomer and resin-based sealants [73]. In the oral cavity, transient drops in pH such as after carbohydrate intake may temporarily enhance fluoride availability and increase enamel resistance during cariogenic episodes [74]. However, frequent acid exposure can compromise surface hardness and increase roughness, particularly in resin-based sealants, potentially reducing long-term retention and become an environment for plaque accumulation [59,75,76,77,78,79,80,81]. This dynamic underscores the importance of pH-cycling and fluoride recharge in maintaining the cariostatic potential of sealants under real intraoral conditions, especially in patients at high caries risk or those with frequent acid challenges.

Most of the reviewed studies reported a biphasic fluoride-release pattern, characterized by an initial phase of rapid ion liberation followed by a gradual decline over time [58,59,60,61,62,63,64]. The early stage corresponds to the diffusion of loosely bound fluoride ions from the outer layer of the material, whereas the later phase reflects the slower migration of ions from deeper layers within the matrix [72,82,83,84,85]. Glass-ionomer and resin-modified glass-ionomer sealants maintained measurable fluoride release for considerably longer periods than resin-based sealants, which demonstrated lower ion availability and shorter release duration [59,61,86,87,88]. These results confirm that fluoride-release kinetics depend on both the intrinsic microstructure of the material and external factors influencing ion transport [29,72,73,82,89,90,91]. While glass-ionomer-based sealants demonstrate superior fluoride release and remineralization capacity, they possess certain drawbacks, including lower mechanical strength, brittleness, and moisture sensitivity, which may limit their long-term retention compared with resin-based systems [92]. Conversely, resin-based sealants provide greater mechanical durability and marginal integrity but exhibit limited fluoride-release potential. Hence, clinical material selection should balance cariostatic benefits with mechanical demands and patient-specific risk profiles [93]. These multifactorial relationships suggest that optimizing both chemical composition and clinical protocols such as regular fluoride recharge could enhance the longevity and effectiveness of sealant therapy.

Interpretation of the present findings must consider several methodological and contextual limitations. The included studies exhibited considerable heterogeneity in sample design, substrate type (human or bovine enamel, standardized blocks), storage media, and analytical techniques, which may have influenced the reported magnitude of fluoride release and complicates direct comparison across experiments. Most studies relied on traditional fluoride ion-selective electrodes, which, while well established, are prone to calibration-dependent variability, limited sensitivity, and only measure free fluoride ions; these shortcomings may affect accuracy and comparability. Moreover, despite advances highlighted in recent literature including methods such as the use of metal-organic frameworks, these innovative methods remain underused in fluoride-release research, limiting methodological modernization [94]. The bulk of the included investigations were conducted under in vitro conditions that simplify the oral environment and fail to replicate variables such as salivary clearance, plaque biofilm, and mechanical wear, thereby limiting external validity. Nevertheless, the overall trends observed across laboratory and clinical studies appear consistent, suggesting that in vitro fluoride-release patterns can still serve as a reasonable approximation of in vivo performance when interpreted cautiously. 

Moreover, restricting the search to English-language publications could have resulted in the omission of relevant data, introducing language bias. The predominance of in vitro studies—often conducted under static or simplified conditions—limits the external validity of the findings, as clinical factors such as salivary flow, biofilm dynamics, and mechanical loading were not replicated. Although a few in vivo investigations provided clinically relevant data, additional long-term randomized clinical trials are needed to evaluate fluoride-release patterns, mechanical stability, and caries-preventive efficacy under true intraoral conditions. An additional limitation is the generally limited information available on the long-term stability or degradation behavior of resin-based sealants. The included studies did not provide sufficiently detailed data to allow for meaningful comparison of materials in this regard. Future research should therefore place greater emphasis on evaluating degradation processes alongside fluoride-release characteristics. Future research should adopt standardized methodologies for fluoride quantification, incorporate dynamic pH-cycling models, and focus on novel bioactive materials, including S-PRG fillers and bioactive glass additives, which have the potential to optimize both ion release and antibacterial performance. Advancing interdisciplinary collaboration between materials science and clinical dentistry will be essential for the development of next-generation bioactive fissure sealants with reliable long-term therapeutic performance.

## 5. Conclusions

Within the limitations of this systematic review, it can be concluded that both the composition of the sealant material and environmental conditions—particularly pH and external fluoride exposure—significantly influence the rate and duration of fluoride ion release. Glass-ionomer and resin-modified glass-ionomer sealants exhibited the highest fluoride release and recharge capacities, confirming their function as long-term fluoride reservoirs capable of maintaining cariostatic ion levels in the oral environment. In contrast, resin-based and giomer sealants released lower amounts of fluoride and showed limited recharge potential, although they demonstrated superior mechanical resistance and lower microleakage. Most studies reported a characteristic biphasic fluoride-release pattern, with an initial burst effect during the first 24–48 h followed by a slower, sustained release phase lasting several weeks or even months. Acidic environments were shown to enhance ion diffusion and surface degradation of glass-ionomer materials, increasing fluoride liberation, while neutral conditions preserved material integrity. The demonstrated ability of some materials to undergo fluoride recharge after topical application of fluoride gels or varnishes highlights the potential clinical benefit of routine professional fluoride treatments in prolonging the cariostatic activity of sealants. From a clinical perspective, the selection of a fluoride-releasing sealant should be individualized, taking into account the patient’s caries risk, moisture control during application, and the need for long-term fluoride availability. Glass-ionomer and resin-modified glass-ionomer sealants are particularly advantageous for patients at high caries risk, in pediatric populations, and in situations where isolation is difficult. In contrast, resin-based sealants remain preferable for low-risk patients due to their superior retention and esthetic properties. Future research should focus on standardized in vivo protocols to better assess fluoride-release mechanisms under dynamic oral conditions and to optimize strategies for maintaining fluoride bioavailability over time.

## Figures and Tables

**Table 1:** General characteristics of studies.

Study	Aim of the Study	Material and Methods	Results	Conclusions
Ei [56]	A comparison of fluoride release and anti-demineralization effects of two resin-based sealants: Teethmate F-1 Sealant, (Kuraray Noritake, Osaka, Japan) and Clinpro Sealant (3M ESPE, St. Paul, MN, USA) and one glass-ionomer sealant Fuji VII (GC, Tokyo, Japan).	Forty bovine enamel samples were divided into four groups and restored with Teethmate F-1, Clinpro, Fuji VII, or a non-fluoride control. Samples were stored in artificial saliva at 37 °C for 14 days, with fluoride release measured every two days. After a 10-day demineralization phase, enamel protection was assessed using SS-OCT and cross-sectional nanohardness testing.	All sealants peaked on day 2, with Fuji VII highest, followed by Teethmate F-1 and Clinpro. Fuji VII released fluoride for 14 days; others for 4 days. Despite lower release, Teethmate F-1 showed the greatest protection against demineralization.	Fuji VII released the most fluoride, but the resin sealant gave better protection against demineralization.
Şişmanoğlu [57]	To compare the fluoride release from four different sealants: BeautiSealant (Shofu, Kyoto, Japan), Clinpro Sealant (3M ESPE, USA), Helioseal F (Ivoclar Vivadent, Schaan, Liechtenstein), Fissurit F (Voco, Cuxhaven, Germany).	Samples of four fissure sealants (BeautiSealant, Clinpro, Helioseal F, Fissurit F) were prepared as disks in Teflon molds, light-cured, and stored in deionized water at 37 °C for 28 days. Fluoride release was measured with a spectrophotometer on days 1, 2, 3, 7, 14, 21, and 28. Deionized water served as control, and tests were performed in triplicate.	All sealants peaked on day 1. BeautiSealant showed a strong burst release that declined within two days, while Clinpro, Fissurit F, and Helioseal F had lower initial values. After one week, fluoride release stabilized and was similar for all materials.	Giomer- and resin-based sealants release most fluoride in the first two days, then stabilize, with giomer-based sealants releasing slightly more initially.
Fita [58]	To evaluate and compare the fluoride release of four fissure sealants: Arkona, Helioseal F (Ivoclar Vivadent), Helioseal F Plus (Ivoclar Vivadent), and Conseal (SDI, Stuart, FL, USA).	Ten discs per material were light-cured for 20 s and stored in deionized water or 0.9% NaCl at 37 °C. Fluoride release was measured up to 2 weeks using a fluoride ion-selective electrode, and materials were characterized by X-ray diffraction (XRD), scanning electron microscopy (SEM), and Fourier transform infrared spectroscopy (FTIR).	Conseal F showed the fastest and highest fluoride release in both media. Peak times varied: Arkona 72 h, Helioseal 24 h (saline) or 2 weeks (water), Helioseal Plus 1–3 h, and Conseal 1 h. Cumulative release ranked Conseal > Helioseal Plus > Helioseal > Arkona. Only Helioseal released more fluoride in saline than in water.	All tested fluoride-containing sealants released fluoride over time, with Conseal showing the highest release, supporting their effectiveness in caries prevention.
Kantovitz [59]	To evaluate how acidic solutions affect the degradation of ionomeric and sealant materials by measuring changes in hardness, surface roughness, and fluoride release.	Three materials were tested: FluroShield (resin sealant), Vitremer (RMGIC), and Ketac Molar (GIC). Eighteen discs per material were polished, and initial surface roughness and Knoop hardness recorded. Specimens were stored for 15 days in 0.3% citric acid, demineralizing solution, or artificial saliva (changed daily). Final roughness, hardness, and daily fluoride release (normalized to surface area) were measured.	All materials peaked on day 1, then declined to stable levels. In citric acid, Vitremer released the most, followed by Ketac Molar and FluroShield. In the demineralizing solution, Vitremer was highest initially, with similar levels after day 3. In artificial saliva, all showed low, stable release.	Ionomeric materials are more vulnerable to acidic conditions, showing greater surface degradation and higher fluoride release, while the resin-based fissure sealant remained stable, with consistent roughness and fluoride release across all solutions.
Prapansilp [60]	To evaluate the effect of varying powder-to-liquid (P/L) ratios in glass-ionomer sealants on fluoride release patterns over time.	4 groups of Fuji VII glass-ionomer specimens (n = 5 each) were prepared with different P/L ratios: control (manufacturer’s ratio), and 25%, 50%, 75% reduced powder. Specimens (3 mm × 5 mm) were light-cured and stored in deionized water at 37 °C. Fluoride release was measured using ion electrode on days 1, 7, 14, and 21.	Fluoride release peaked on day 1, stabilized by day 7, and remained constant for 21 days. Materials with 50–75% less powder released significantly more fluoride than control and 25–reduced groups (*p* < 0.05). Lower powder-to-liquid ratios increased fluoride release throughout.	GI sealants with reduced P/L ratios released significantly more fluoride than manufacturer recommended ratios. Lower ratios enhance fluoride release.
Asvanund [61]	The study compared fluoride release and recharge ability of a glass ionomer and a resin-based sealant after exposure to acidulated phosphate fluoride gel.	This in vitro study used three materials: a non-fluoride resin sealant (Concise), a fluoride-releasing resin sealant (Clinpro), and a glass ionomer sealant (Fuji VII). For each type, 15 disc-shaped samples (11 mm × 1 mm) were made. Fluoride release was measured over 21 days, then all samples were exposed to 1.23% APF gel for 4 min. Post-treatment fluoride release was measured for 5 more days.	The glass ionomer released much more fluoride than the resin sealant (6.62 vs. 0.41 ppm on Day 1; 0.55 vs. 0.02 ppm on Day 21). After APF gel exposure, release peaked again (15.42 vs. 0.16 ppm), while the control showed none.	The glass ionomer sealant released more fluoride and showed greater recharge ability after APF gel exposure than the resin-based sealant.
Dionysopoulos [62]	The aim of this in vitro study was to assess how three newly developed fissure sealants perform in terms of fluoride release and their potential to be recharged.	The study tested three fluoride-releasing sealants (Teethmate F-1, Fissurit F, BeautiSealant) and a glass ionomer cement (FX-II) as control. Eight specimens of each were prepared, stored in deionized water, and fluoride release was measured for 28 days using an ion-selective electrode. Afterward, samples were immersed in 0.05% NaF solution for 5 min to assess re-release over 5 days.	Over 28 days, fluoride release ranked FX-II > Teethmate F-1 > Fissurit F > BeautiSealant. After 0.05% NaF re-fluoridation, FX-II again showed the highest re-release, with all sealants releasing significantly less (*p* < 0.05).	The sealants showed varying fluoride release and recharge abilities, but all were much less effective than the glass ionomer.
Poggio [63]	The aim of the study was to evaluate and compare the fluoride release and fluoride uptake abilities of different fissure sealants, specifically a glass ionomer cement (Fuji Triage) and two resin-based sealants (Fissurit FX, Grandio Seal).	Three sealants—Fuji Triage, Fissurit FX, and Grandio Seal—were tested for fluoride release in deionized water at 37 °C for 49 days. After 7 weeks, samples were treated with fluoride varnish or CPP-ACP toothpaste to assess fluoride uptake and re-release.	The glass ionomer released significantly more fluoride than resin sealants (*p* < 0.001). After day 21, Fissurit FX exceeded Grandio Seal. Profluorid Varnish enhanced fluoride release in all materials, while MI Paste Plus had a weaker effect.	The glass ionomer sealant showed the highest fluoride release initially and after re-fluoridation. Resin sealants released less but responded to fluoride varnish, with Profluorid Varnish more effective than CPP-ACP toothpaste.
Ananda [64]	The aim of the study was to evaluate and compare the fluoride release into dental plaque from fluoride-releasing resin-based and glass ionomer sealants applied using the ART approach, assessed at different time intervals.	A randomized clinical trial on 60 children divided into four groups (Teethmate-F1, Helioseal-F, Fuji IX GP, Ketac-Molar; n = 15 each). Non-fluoridated toothpaste was used; diet and oral hygiene were controlled. Plaque samples were collected at baseline, 24 h, 9 days, 2 and 4 weeks, and fluoride concentration was measured with an ion-specific electrode after enzymatic hydrolysis.	Baseline plaque fluoride levels did not differ significantly. All sealants peaked at 24 h, with Fuji IX highest, followed by Ketac Molar. Significant differences occurred at 24 h, 9 days, 2 weeks, and 1 month (*p* < 0.05). Resin sealants released less fluoride and for a shorter duration than glass ionomers.	All materials released fluoride for 4 weeks, with an initial burst followed by sustained release. Glass ionomers released more than resin sealants. Salivary fluoride returned to baseline within a month, while plaque fluoride stayed significantly elevated.
Fan [65]	This study aimed to develop and evaluate experimental antibacterial fluoride-releasing sealants and to compare their fluoride release, recharge potential, adhesion, and microleakage with those of commercial sealants.	Two experimental fluoride-releasing sealants (Exp-1 with 15% NovaMin, Exp-2 without) were compared with FluroShield, Clinpro, and SeLECT Defense. Discs (5 mm × 1.2 mm, n = 5) were light-cured and stored in deionized water at 37 °C. Fluoride release was measured daily for 14 days, then after three 2% NaF recharge cycles.	Experimental sealants released and recharged more fluoride than commercial ones (*p* < 0.05). Exp-2 showed higher release at some points, Exp-1 greater net recharge. Both had significantly lower microleakage (*p* < 0.05).	Experimental sealants showed superior fluoride performance and sealing ability, warranting further clinical evaluation.
Kaga [66]	The aim of this study was to evaluate the properties of pit and fissure sealants containing functional fillers by assessing their ion release and diametral tensile strength.	Four sealants—S-FS, Delton FS+, Teethmate F-1 2.0, and Fuji III LC—were tested. Disc specimens (n = 72) were light-cured and stored in distilled water at 37 °C. Diametral tensile strength was measured after 24 h, 4, and 12 weeks; fluoride release weekly for 12 weeks. Ion release was analyzed by ICP-AES, and fractures examined by SEM.	All sealants released fluoride, peaking in week 1 then stabilizing. Fuji III LC showed the highest release, followed by Delton FS+, S-FS, and Teethmate F-1 2.0. ICP confirmed characteristic ion release, and tensile strength remained stable, higher for S-FS and Delton FS+ than for Teethmate F-1 and Fuji III LC (*p* < 0.05).	The S-PRG sealant showed high strength and steady fluoride release. Its strontium and boron ions may aid antibacterial action and remineralization, acting as a slow-release, pH-buffering system against caries.
Koga [67]	Analysis of fluoride-containing fissure sealants on caries prevention.	Four sealants—Fuji III, Fuji III LC, Teethmate F-1, and Helioseal F—were tested for fluoride release and recharge. Discs were stored in distilled water at 37 °C for 7 days, treated with APF, then re-immersed for 14 days. Fluoride release and Fuji III LC fluoride uptake were measured.	Fuji III showed the highest fluoride release, while Fuji III LC had the greatest recharge and higher enamel uptake after APF treatment. Helioseal F and Teethmate F-1 showed minimal fluoride recharge.	Fissure sealants containing GIC serve as fluoride reservoir in the oral cavity, therefore deliver a constant low fluoride level, which acts anticariogenic.
Kusgöz [68]	The study evaluated nano-filled resin sealant Grandio Seal for degree of conversion, microhardness, microleakage, and fluoride release, comparing it with Clinpro (resin) and Fuji Triage (glass ionomer).	Disk-shaped specimens from fissure sealants were tested for degree of conversion (DC), Vickers hardness (VHN) after 24 h, and cumulative fluoride release (FR) 1 h, 6 h, 12 h, 1, 7, 15 and 30 days. Microleakage evaluation: Sealants were applied on etched enamel of third molars (n = 10), followed by thermocycling and mechanical loading before assessing microleakage.	Degree of conversion: FT (89%) > GS (55.02%) > CL (%51.10) (*p* < 0.05). Vicker hardness: GS > FT > CL (*p* < 0.05). Fluoride release was significantly higher in FR compared to others (*p* < 0.05). Higher microleakage scores were observed in FT (*p* < 0.05).	Nano-filled resin based sealant has superior hardness results and feasible sealing ability, therefore can serve as an alternative to other sealants.
Kamala [69]	Comparison of Fuji III and Fuji VII glassionomer sealants as to their retention, caries incidence and salivary fluoride release between two groups of children (6 year olds and 8 year olds).	Healthy first permanent molars of 110 children aged 6–8 were randomly assigned to two groups: Fuji VII (A) and Fuji III (B), with opposing molars serving as untreated controls. Children with high caries risk or prior fluoride exposure were excluded. Evaluations were performed after 24 h, 7 days, and 1, 3, 6, and 12 months, assessing sealant retention by probing, caries incidence visually, and fluoride release from saliva samples collected from seven randomly selected children per group using an Orion ion analyzer with a fluoride-selective electrode.	Both sealants showed 100% retention at 1 month, decreasing to 16.4% loss for Fuji VII and 20% for Fuji III after 1 year. No caries developed in sealed or control teeth. Salivary fluoride peaked at 24 h (0.092 ppm Fuji VII, 0.104 ppm Fuji III) and declined by day 7. Significant correlations were found between groups at 1 and 12 months, and for Fuji VII at 24 months, with no significant differences between sealants over time.	After 12 months, over 75% of teeth showed partial or total retention with no difference between sealants. No caries occurred, and fluoride release followed a consistent pattern—initial burst, decline, then gradual return to baseline.
Lobo [70]	The study evaluated three sealants—RMGIC, fluoride-releasing composite, and non-fluoridated composite—for their cariostatic effect, including barrier action, fluoride protection, and distant enamel uptake.	Forty-eight extracted third molars were divided into four groups: control, RMGIC, fluoride-releasing composite, and non-fluoridated composite. Groups 2–4 underwent 5-day pH cycling; controls were stored moist at 37 °C. Fluoride release, uptake, and enamel demineralization were assessed by ion analysis, biopsy, and microhardness testing.	The RMGIC released more fluoride, increased enamel uptake, and reduced demineralization most effectively. The fluoride-releasing composite had moderate effects, while the non-fluoridated composite showed none.	The RMGIC sealant showed superior fluoride release, enamel uptake, and protection against demineralization, offering added cariostatic benefits for high-risk patients
Leao [71]	The study evaluated a bioactive S-PRG resin composite for its ability to inhibit enamel demineralization compared to conventional and resin-modified glass-ionomers.	Study was performed in vitro, ninety bovine enamel blocks were divided into 6 groups of 15 samples: Fuji IX, Ion Z, Fuji II LC, Beautifil II (S-PRG composite), Filtek Z250 (composite resin) and control group without any treatment performed. Cavities were restored, then subjected to a 7-day pH cycling regimen (demineralization/remineralization). Surface hardness was measured and enamel was analyzed by energy dispersive X-ray spectroscopy.	Glass ionomers showed the highest post-cycling hardness. The S-PRG composite had intermediate values and uniquely increased enamel fluoride while reducing calcium content.	The S-PRG composite partly inhibited demineralization and enhanced fluoride uptake but was less remineralizing than glass ionomers, warranting further in vivo study.

**Table 2:** Detailed characteristics of included studies.

Study	Study/Samples Design	Fluoride Sealants	Storage Conditions	Measurement Time and Method	Total Fluoride Released	Additional Findings
Ei [56]	In vitro, 20 bovine incisors, each divided into two enamel blocks	-Teethmate F-1 Sealant, Kuraray Noritake, Osaka, Japan, -ClinproTM Sealant 3M ESPE, St. Paul, MN, USA -GC Fuji VII, GC	0.5 mL of artificial saliva (1 mM CaCl_2_, 3 mM H_2_PO_4_, 100 mM NaCl, 100 mM Na acetate, 0.02% NaN_3_, salivary phosphoprotein homologue Casein 100 µg/mL), pH-6.3, 37 °C; specimens were transferred to new solution every 2 days for 14 days	Measurements taken every two days for 14 days. Measurement method: ion meter (F-53, Horiba, Kyoto, Japan).	All sealants peaked on day 2, with Fuji VII releasing the most fluoride, followed by Teethmate F-1 and Clinpro. Fuji VII maintained release throughout 14 days, while the others declined after day 4. Cumulative release was highest for Fuji VII (69.5 µg/cm^2^), then Teethmate F-1 (7.26 µg/cm^2^) and Clinpro (3.94 µg/cm^2^).	Teethmate F-1 showed the greatest resistance to demineralization. Fuji VII and Clinpro demonstrated moderate, comparable protection, which was not significantly different from each other or the control after 10 days.
Şişmanoğlu [57]	In vitro, 28 disc samples (5 mm diameter, 2 mm thickness)	-BeautiSealant (Shofu, Japan), -Clinpro Sealant (3M ESPE, USA), -Helioseal F (Ivoclar Vivadent, Liechtenstein), -Fissurit F (Voco, Germany)	5 mL of deionized water, 37 °C	Measurement on day 1, 2, 3, 7, 14, 21, and 28 Measurement method: spectrophotometer (Thermo Scientific Evolution 160 UV-VIS, Bremen, Germany)	BeautiSealant [ppm] Day 1: 5.33 ± 0.67 Day 2: 2.17 ± 0.27 Day 3: 1.85 ± 0.28 Day 7: 1.59 ± 0.21 Day 14: 1.29 ± 0.06 Day 21: 1.16 ± 0.02 Day 28: 1.12 ± 0.02 Clinpro Sealant [ppm] Day 1: 2.69 ± 0.43 Day 2: 2.90 ± 0.22 Day 3: 1.86 ± 0.30 Day 7: 1.11 ± 0.07 Day 14: 1.07 ± 0.09 Day 21: 0.96 ± 0.04 Day 28: 1.00 ± 0.0 Heliosel F [ppm] Day 1: 2.91 ± 0.64 Day 2: 2.84 ± 0.38 Day 3: 2.00 ± 0.24 Day 7: 1.25 ± 0.16 Day 14: 1.22 ± 0.07 Day 21: 1.24 ± 0.03 Day 28: 1.01 ± 0.03 Fissurit F [ppm] Day 1: 2.94 ± 0.67 Day 2: 2.90 ± 0.16 Day 3: 2.15 ± 0.08 Day 7: 1.26 ± 0.04 Day 14: 1.15 ± 0.08 Day 21: 1.18 ± 0.01 Day 28: 1.21 ± 0.03	n/a
Fita [58]	In vitro, 10 disc samples (4 mm diameter, 2 mm thickness)	-Helioseal F (Ivoclar Vivadent), -Helioseal F Plus (Ivoclar Vivadent), -Conseal F (SDI) -Arkona	Deionized water (5 samples) and 0.9 % NaCl (5 samples), both 37 °C.	Measurement after 1, 3, 24, 48, 72, and 96 h, and after 1 and 2 weeks Measurement method: ion-selective electrode (ORION 9609)	0.9% NaCl: Arkona [ppm/mg] 1 h: 0.0004 ± 0.0001 3 h: 0.0008 ± 0.0003 24 h: 0.0008 ±0.0001 48 h: 0.0009 ± 0.0002 72 h: 0.0012 ± 0.0002 96 h: 0.0006 ± 0.0001 1 week: 0.0008 ± 0.0002 2 weeks: 0.0007 ± 0.0005 Helioseal F [ppm/mg] 1 h: 0.0009 ± 0.0001 3 h: 0.0008 ± 0.0001 24 h: 0.0013 ± 0.0003 48 h: 0.0009 ± 0.0001 72 h: 0.0011 ± 0.0001 96 h: 0.0006 ± 0.0001 1 week: 0.0008 ± 0.0001 2 weeks: 0.0011 ± 0.0002 Conseal [ppm/mg] 1 h: 0.0051 ± 0.0018 3 h: 0.0023 ± 0.0006 24 h: 0.0022 ± 0.0003 48 h: 0.0025 ± 0.0010 72 h: 0.0016 ± 0.0006 96 h: 0.0011 ± 0.0006 1 week: 0.0009 ± 0.0002 2 weeks: 0.0011 ± 0.0005 Helioseal F Plus [ppm/mg] 1 h: 0.0017 ± 0.0007 3 h: 0.0016 ± 0.0004 24 h: 0.0015 ± 0.0004 48 h: 0.0014 ± 0.0003 72 h: 0.0009 ± 0.0006 96 h: 0.0007 ± 0.0002 1 week: 0.0007 ± 0.0002 2 weeks: 0.0014 ± 0.0006 Deionized water: Arkona [ppm/mg] 1 h: 0.0005 ± 0.0002 3 h: 0.0006 ± 0.0002 24 h: 0.0007 ± 0.0001 48 h: 0.0013 ± 0.0004 72 h: 0.0009 ± 0.0002 96 h: 0.0005 ± 0.0001 1 week: 0.0007 ± 0.0002 2 weeks: 0.0010 ± 0.0005 Helioseal F [ppm/mg] 1 h: 0.0005 ± 0.0001 3 h: 0.0007 ± 0.0004 24 h: 0.0008 ± 0.0004 48 h: 0.0006 ± 0.0002 72 h: 0.0007 ± 0.0002 96 h: 0.0005 ± 0.0001 1 week: 0.0007 ± 0.0001 2 weeks: 0.0010 ± 0.0003 Conseal [ppm/mg] 1 h: 0.0041 ± 0.0014 3 h: 0.0019 ± 0.0007 24 h: 0.0017 ± 0.0002 48 h: 0.0016 ± 0.0003 72 h: 0.0012 ± 0.0003 96 h: 0.0007 ± 0.0002 1 week: 0.0009 ± 0.0001 2 weeks: 0.0012 ± 0.0005 Helioseal F Plus [ppm/mg] 1 h: 0.0012 ± 0.0002 3 h: 0.0016 ± 0.0009 24 h: 0.0015 ± 0.0005 48 h: 0.0015 ± 0.0005 72 h: 0.0005 ± 0.0001 96 h: 0.0004 ± 0.0001 1 week: 0.0007 ± 0.0001 2 weeks: 0.0013 ± 0.0004	n/a
Kantovitz [59]	In vitro, 54 disc samples (6 mm diameter, 2 mm thickness)	-FluroShield, (Dentsply DeTrey, Konstanz, Germany) -Vitremer (3M ESPE, St. Paul, MN, USA) -Ketac Molar (3M ESPE, St. Paul, MN, USA)	- 0.3% citric acid solution, pH 3.2 - demineralizing solution (2.0 mM calcium, 2.0 mM phosphate and acetate buffer 75 mM), pH 4.3 - artificial saliva (1.5 mM calcium, 0.9 mM phosphate, KCl 150 mM and Tris [tris- (hydroxymethyl) aminomethane] buffer 20 mM), pH 7.0 All solutions of 3 mL and 25 °C	Measurement after 1, 2, 3, 5, 7, 9, 12, 15 days Measurement method ion- selective electrode (Orion 96-09; Orion Research Inc., Boston, MA, USA), digital ion-analyzer (Orion EA- 940; Orion Research Inc.)	All materials showed a day-1 fluoride burst, stabilizing by day 15. Vitremer released the most in demineralizing and citric acid solutions, while Ketac Molar dominated later. FluroShield released the least in all media.	Citric acid caused the greatest roughness increase for Vitremer and Ketac Molar, while FluroShield was less affected. Initially, Ketac Molar was hardest, Vitremer intermediate, and FluroShield softest. All materials showed significant hardness loss in all solutions.
Prapansilp [60]	In vitro study, sample: 3 mm × 5 mm blocks prepared in plastic molds 20 samples total, divided into 4 groups of 5 samples each	Fuji VII (GC Corp, Tokyo, Japan)—GI sealant Preparation: manual mixing of powder and liquid with light curing for 20 s on both sides	deionized water (10 mL per sample), 37 °C	Time points: days 1, 7, 14, 21 Method: Fluoride-specific ion electrode (Orion EA940 expandable) connected to digital ion analyzer (Orion 96-09) Sample preparation: 10 mL sample + 1 mL TISAB III buffer, stirred for 3 minutes	Day 1 concentrations (highest release): - group 1 (manufacturer ratio): 8.27 ± 0.56 ppm - group 2 (25% less powder): 3.2 ± 0.52 ppm - group 3 (50% less powder): 1.51 ± 0.14 ppm - group 4 (75% less powder): 1.36 ± 0.30 ppm Ranking by total fluoride release: Group 1 > Group 2 > Group 3, Group 4 Pattern: All groups showed highest release on day 1, then decreased by day 7 and remained constant through day 21.	Fluoride release peaked on day 1 from loosely bound ions, then stabilized after day 7 through diffusion. Lower powder-to-liquid ratios increased fluoride solubility and release.
Asvanund [61]	In vitro study; 45 disc samples total (15 per sealant), each 11 mm × 1 mm	Concise (resin, no fluoride, control); Clinpro (resin, fluoride-releasing); Fuji VII (glass ionomer, fluoride-releasing)	10 mL deionized water, replaced daily, 37 °C	Fluoride concentration was measured on Days 1, 2, 3, 4, 5, 6, 7, 14 and 21. After 21 days, samples were immersed for 4 minutes in 1.23% acidulated phosphate fluoride (APF) gel and then returned to fresh deionized water. Post-exposure measurements were taken for 5 additional days. Fluoride was measured using a fluoride ion-selective electrode after mixing with TISAB III buffer.	Day 1: Fuji VII: 6.62 ± 2.07 ppm Clinpro: 0.41 ± 0.06 ppm Concise: 0.00 ± 0.00 ppm Day 21: Fuji VII: 0.55 ± 0.31 ppm Clinpro: 0.02 ± 0.00 ppm Concise: 0.00 ± 0.00 ppm After APF gel recharge (Day 22): Fuji VII: 15.42 ± 2.48 ppm Clinpro: 0.16 ± 0.02 ppm Concise: 0.10 ± 0.03 ppm Day 26 (5 days post-recharge): Fuji VII: 1.24 ± 0.31 ppm Clinpro: 0.02 ± 0.00 ppm Concise: 0.01 ± 0.00 ppm Glass ionomer (Fuji VII) consistently released the highest amounts of fluoride, both initially and after recharging, compared with resin-based materials.	All materials recharged after APF exposure, most notably the glass ionomer, whose porous structure enabled greater fluoride uptake than resin sealants.
Dionysopoulos [62]	In vitro study; 8 cylindrical specimens (7 mm × 2 mm) for each material	Teethmate F-1 (Kuraray), Fissurit F (Voco), BeautiSealant (Shofu); Control: FX-II glass ionomer (Shofu)	5 mL de-ionized water at 37 °C	Fluoride release measured daily (days 1–7), then weekly up to 28 days; method: fluoride ion-selective electrode. After 28 days → specimens recharged in 0.05% NaF (5 min) and re-release measured daily for 5 days	Total Fluoride Released (28 days) FX-II: 408.6 ± 45.66 µg/cm^2^ > Teethmate F-1: 89.45 ± 12.32 µg/cm^2^ > Fissurit F: 68.62 ± 8.72 µg/cm^2^ > BeautiSealant: 33.32 ± 4.91 µg/cm^2^ (*p* < 0.05) Fluoride Re-release (5 days after recharge) FX-II: 99.53 ± 13.21 µg/cm^2^ > Teethmate F-1: 9.76 ± 1.62 µg/cm^2^ > BeautiSealant: 5.69 ± 0.89 µg/cm^2^ > Fissurit F: 4.76 ± 0.72 µg/cm^2^ (*p* < 0.05)	Glass ionomer (FX-II) showed the greatest fluoride release and recharge. Among resin sealants, Teethmate F-1 released the most fluoride, while BeautiSealant had the highest recharge potential
Poggio [63]	In vitro study, 3 groups, 10 specimens each (n = 30).	Fuji Triage (GIC, GC); Fissurit FX (resin + 3% NaF, Voco); Grandio Seal (resin, no fluoride, Voco).	3 mL deionized water, 37 °C, solution renewed.	Fluoride release was measured on days 1, 2, 3, 5, 7, 21, 35, and 49. After 7 weeks, the specimens were treated with fluoride varnish (Profluorid Varnish) or CPP-ACP paste with fluoride (MI Paste Plus), and re-release was measured on days 56, 70, and 84. Fluoride concentration was determined using a fluoride ion-selective electrode connected to an ion analyzer.	Fuji Triage (GIC) released the highest amount of fluoride throughout the experiment: from 1.1 ppm on day 1 up to 8.0 ppm on day 49. Fissurit FX (resin + NaF) released smaller amounts: 0.10 ppm on day 1, increasing to 1.03 ppm on day 49. Grandio Seal (resin, no fluoride) showed almost negligible release: 0.02 ppm on day 1 to 0.04 ppm on day 49. Ranking: Fuji Triage ≫ Fissurit FX > Grandio Seal	All sealants demonstrated the ability to absorb fluoride from external sources and re-release it.. Profluorid Varnish (5% NaF) strongly increased fluoride release in all materials: e.g., Fuji Triage from 8.0 ppm (day 49) to 61.3 ppm (day 84); Fissurit FX up to 48.5 ppm; Grandio Seal up to 57.3 ppm. MI Paste Plus (CPP-ACP + fluoride) showed a weaker effect. It increased fluoride release slightly in Fissurit FX and Grandio Seal (≤1.4 ppm), but was ineffective in Fuji Triage (≤0.13 ppm). Fluoride varnish proved significantly more effective than MI Paste Plus in recharging sealants.
Ananda [64]	In vivo, comparative clinical trial; 60 schoolchildren from Davangere, randomized into 4 groups (n = 15 each).	- Group I: Teethmate-F1 (methacryloyl methyl methacrylate sealant) - Group II: Helioseal-F (sealant with fluorosilicate glass) - Group III: Fuji IX GP (high-viscosity glass ionomer, ART) - Group IV: Ketac-Molar (high-viscosity glass ionomer, ART)	Non-fluoridated dentifrice provided; subjects instructed to refrain from other oral hygiene procedures, avoid high-fluoride foods, and abstain from tea during the study.	Plaque collected at baseline (3 days, mean) and after sealant application (24 h, 9 days, 2 weeks, 4 weeks) from first permanent molars (curette); fluoride analyzed after phosphatase incubation (37 °C) using ion-selective electrode (Orion 94-09) with TISAB.	Helioseal F [ppm]: Baseline: 32.79 ± 2.32 After 24 h: 84.47 ± 5.13 After 9 days: 51.50 ± 7.9 After 2 weeks: 38.74 ± 5.16 After 1 month: 34.06 ± 3.0 Teethmate F1 [ppm]: Baseline: 33.42 ± 3.31 After 24 h: 72.83 ± 4.75 After 9 days: 47.21 ± 5.0 After 2 weeks: 34.45 ± 4.55 After 1 month: 31.39 ± 4.8 Fuji IX GP [ppm]: Baseline: 31.93 ± 4.68 After 24 h: 103.78 ± 11.8 After 9 days: 87.55 ± 6.3 After 2 weeks: 55.72 ± 14.1 After 1 month: 40.66 ± 6.6 Ketac Molar [ppm]: Baseline: 32.85 ± 3.98 After 24 h: 97.96 ± 8.89 After 9 days: 77.91 ± 7.0 After 2 weeks: 55.32 ± 5.98 After 1 month: 39.05 ± 6.1	No statistically significant differences were observed in salivary pH (range 7.11–7.33) or dmfs index (5.2–6.1) among groups at baseline (*p* = 0.100).
Fan [65]	In vitro study; disk specimens (5.0 mm × 1.2 mm; n = 5 per group) prepared from experimental and commercial sealants and light-cured for 40 s with Optilux 501 curing unit.	- Exp-1: antibacterial fluoride-releasing monomer, hydrolytically stable adhesive monomer, other dental monomers, fluoride-releasing glass filler + 15% NovaMin bioactive glass nanoparticles. - Exp-2: same as Exp-1 but without NovaMin. - FluroShield (FS; Caulk/Dentsply) – commercial fluoride-releasing sealant. - Clinpro (CP; 3M ESPE) – fluoride-releasing antimicrobial sealant. - SeLECT Defense (E34; Element 34 Technology)—antibacterial, non-fluoride-releasing sealant.	Specimens were immersed individually in 2.0 mL deionized water at 37 °C; immersion solution refreshed daily.	Fluoride release measured daily for 14 days using ion-selective electrode (Orion 96-09, Thermo Scientific, Waltham, MA, USA) with TISAB buffer and 720 pH/ISE meter. After 2 additional days of release (baseline), specimens were recharged with 2.0% NaF gel (Neutra-Foam, 1 min), rinsed 30 s, and fluoride release measured daily for 4 days. Recharge cycle repeated 3 times; data from the 4th day after each recharge served as new baseline.	Exp-1 [µg/cm^2^]: Cumulative fluoride release in 14 days: 23.90 ± 2.85 Cumulative fluoride release 3 days after recharge: 2.93 ± 0.61 Exp-2 [µg/cm^2^]: Cumulative fluoride release in 14 days: 45.34 ± 4.35 Cumulative fluoride release 3 days after recharge: 6.44 ± 0.72 Clinpro [µg/cm^2^]: Cumulative fluoride release in 14 days: 4.98 ± 0.97 Cumulative fluoride release 3 days after recharge: 0.46 ± 0.05 FluroShield [µg/cm^2^]: Cumulative fluoride release in 14 days: 4.79 ± 1.10 Cumulative fluoride release 3 days after recharge: 0.56 ± 0.13	Both experimental and commercial sealants showed comparable bonding strength to enamel at 24 h and after thermocycling (*p* > 0.05). The experimental sealants exhibited little to no microleakage, which was significantly lower than that of the commercial products, indicating superior sealing performance.
Kaga [66]	In vitro study: 24 disc specimens (6 mm × 3 mm) per sealant were prepared. Resin sealants were light-cured 30 s per side plus 30 s post-removal, and Fuji III LC was mixed and cured similarly.	- S-FS: resin-based fissure sealant with S-PRG fillers (Shofu Inc.) - Delton FS+ (DE): resin-based sealant containing barium alumino-fluorosilicate glass fillers - F-1 2.0 (TF): resin-based sealant with fluoroaluminosilicate glass fillers - Fuji III LC (III LC): resin-modified glass ionomer cement sealant containing fluoroaluminosilicate glass fillers and polyacrylic acid	Specimens incubated individually at 37 °C in 3 mL of distilled water; immersion medium changed weekly.	Fluoride release measured weekly for 12 weeks; specimens rinsed, dried, incubated in 3 mL distilled water at 37 °C. Fluoride concentration analyzed with ion-selective electrode (Orion 9609 BNWP) + TISAB III.	- S-FS: 6.1 [ppm] - DL (Delton FS+): 9.2 [ppm] - TF (F-1 2.0): 2.7 [ppm] - III LC (Fuji III LC): 15.8 [ppm]	S-FS and Delton showed stable, significantly higher DTS than TF and Fuji III LC (*p* < 0.05), with Fuji III LC lowest. S-FS released notable strontium, boron, and fluoride ions, suggesting antibacterial potential.
Koga [67]	In vitro study, n = 20 (5 disks were shaped from each sealant); 5 bovine anterior teeth (measuring fluoride uptake)	Fuji III, Fuji III LC (GC), Teethmate F-1 (Kuraray) and Helioseal F (Vivadent)	Each disk was stored in 5 mL distilled water at 37 °C, with water changed daily. After 7 days, disks were treated with APF for 4 min, rinsed, and re-immersed in distilled water for another 14 days.	Fluoride ions release from the sealants was analyzed by fluoride ion-selective electrode (Model 96-09BN, Orion Research Co.) connected to an ion-analyzer (EA920, Orion Research Co.) every day of the experiment..	Up to day 7, the highest cumulative fluoride release was from FIII (23,745.2 µg/cm^2^), while FIII L and TF1 showed much lower but similar levels (≈53 µg/cm^2^). HSF had the lowest release (3.7 ± 2.8 µg/cm^2^). On day 1, FIII released 71.9 ± 12.6 µg/cm^2^/day, dropping to 19.3 ± 3.3 µg/cm^2^/day by day 5. For FIII L and TF1, the initial release was ≈ 17.2–18.4 µg/cm^2^/day, decreasing to one-third (≈4.0–4.7 µg/cm^2^/day) by day 4. FIII showed the highest initial and cumulative release, but FIII L performed best after fluoride recharge.	After APF on day 7, only GIC sealants (Fuji III, Fuji III LC) showed recharge, higher in Fuji III LC (74.1 µg/cm^2^) than Fuji III (58.7 µg/cm^2^), lasting about one day. Enamel fluoride was significantly higher in both groups vs. controls (*p* < 0.05), with Fuji III LC showing slightly greater uptake across all layers.
Kusgöz [68]	In vitro n = 45 (15 disks from each sealant; 5 disks from each group were used in 1 of 3 tests)	Clinpro, 3M ESPE Fuji Triage GC Grandio Seal, VOCO	distilled water, 37 °C for 24 h before testing	Cumulative fluoride was measured electrochemically with a fluoride ion-selective (ELIT 8221) and Ag/AgCl reference electrode using 5 mL test solution plus 0.5 mL TISAB. Measurements were taken at 1 h, 6 h, 12 h, 1, 7, 15, and 30 days with solution renewal at each interval.	Concentration after sequentially 1 h; 6 h; 12 h; 1 day; 4 day; 7 day;15 day; 30 day: - Clinpro: 0.74 ± 0.16; 1.29 ± 0.20, 1.48 ± 0.22, 6.47 ± 0.07; 23.67 ± 0.53; 43.37 ± 4.49; 54.33 ± 4.52; 58.18 ± 4.08 -Fuji Triage: 106 ± 1.58; 347.9 ± 2.3; 517.4 ± 1.32; 957.2 ± 4.45; 1419 ± 15.3; 1884.9 ± 18.7; 2276.8 ± 28.1; 2698 ± 22 -Grandio Seal: 0.38 ± 0.01; 0.73 ± 0.02; 0.95 ± 0.11; 4.56 ± 0.18; 20.97 ± 0.83; 35.16 ± 3.07; 43.96 ± 0.82; 47.83 ± 1.7. Fuji Triage > Clinpro > Grandio seal	Degree of conversion%: Fuji Triage > Grandio Seal > Clinpro Microhardness: Grandio Seal > Fuji Triage > Clinpro Microleakage: Clinpro > Grandio Seal > Fuji Triage
Kamala [69]	In vivo, n = 110 molars of children aged 6–8	Fuji III, Fuji VII	Oral cavity	Stimulated whole saliva samples were collected from 7 children of each groups (chosen randomly) at 24 h, 7 days, 1, 3, 6 and 12 months, analysis was carried out by an Orion microprocessor ion analyzer with a fluoride-specific ion electrode	Fluoride concentration in saliva [ppm] 0 h Baseline 0.078 ppm 24 h Fuji VII 0.092 Fuji III 0.104 7 days Fuji VII 0.058. Fuji III 0.059 1 month Fuji VII 0.087 Fuji III 0.094 3 months Fuji VII 0.082 Fuji III 0.085 6 months Fuji VII 0.075 Fuji III 0.082 12 months Fuji VII 0.074 Fuji III 0.083	Retention on clinical visual inspection: total or partial retention of Fuji VII and Fuji III sealants-100% until the 1-month. Total loss at the 12-month recall: 16.4% for Fuji VII, 20% for Fuji III. No caries on sealed and unsealed molars for 12 months.
Lobo [70]	In vitro, 48 extracted human third molars (impacted, without caries) divided into 4 groups of n = 12 G1- without sealant (control) G2- resin-modified glass ionomer G3- fluoride-releasing composite sealant G4- non-fluoridated composite sealant	-Vitremer (3M ESPE)-RMGI -Clinpro Sealant (3M ESPE)-FRCS -Concise (3M ESPE)-NFCS	Group 1: moist environment, 37 C Groups 2–4: subjected to 5-day pH cycling model simulating caries challenge: -6 h/day in demineralizing solution (2 mM Ca, 2 mM PO4, 0.075 M acetate buffer, pH 4.3) 18 h/day in remineralizing solution (1.5 mM Ca, 0.9 mM PO4, 150 mM KCl, 0,1 M Tris buffer, pH 7.0) Solutions were replaced daily	- Fluoride release: pooled daily De+Re solutions, measured by ion-selective electrode (Orion 96-09) with TISAB III standards (0.025–2.0 µg/mL). - Fluoride uptake: enamel biopsy on buccal window (4 mm^2^), sequential HCl etching (15, 30, 60 s), fluoride measured with ion-selective electrode + TISAB II. - Microhardness: cross-sectional microhardness (Knoop, 25 g/5 s), indents at 10, 20, 30, 40, 50 µm below & above sealant margin. Mineral content calculated from KHN.	Cumulative fluoride release over 5 days (µg F/ml, mean ± SD): - Vitremer (RMGI): 1.65 ± 0.13 - Clinpro (FRCS): 0.33 ± 0.03 - Concise (NFCS): 0.27 ± 0.03 - Control: 0.00 Fluoride uptake (µg F/cm^2^ in first enamel layer): - RMGI: ~9.4 ± 1.5 - FRCS: ~4.3 ± 1.2 - NFCS: ~3.6 ± 0.9 - Control: ~2.7 ± 0.8	Enamel was sealed- all materials prevented demineralization -Adjacent enamel (10–20 µm depth): RMGI significantly reduced demineralization compared to other groups -Clinpro had a slight cariostatic effect in distant enamel; however, it was weaker than that of RMGI -Concise had no cariostatic effect -RMGI presented highest fluoride uptake
Leao [71]	In vitro, 90 bovine enamel blocks (size: 4 mm × 4 mm × 2 mm) divided into 6 groups: -NT: no treatment applied. Control group -F IX-Fuji IX Extra (conventional GIC) -IZ Ion Z (conventional GIC) F II-Fuji II LC (resin-modified GIC) B II-Beautiful II (S-PRG giomer composite) F250-Filtek Z250 XT (non-fluoridated resin, negative control group)	Fuji IX Extra (conventional GIC) Ion Z (conventional GIC) Fuji II LC (RMGIC) Beautiful II (giomer composite) Filtek Z250 XT (Non fluoridated composite)	7-day dynamic pH cycling at 37 C: 6 h/day in demineralizing (2.0 mM Ca; 2.0 mM P acetate, 0.02 ppm F, pH 4.7) and 18 h/day in remineralizing solution (1.5 mM Ca; 0.9 mM P, 150 mM KCl, 0.03 ppm F, pH 7.0)	Surface hardness (Knopp, 25 g/10 s) at baseline and after cycling, measured in distance of 150/300/450 µm from restoration margins. Elemental analysis of enamel by SEM-EDX. No direct measurement of fluoride in storage solutions	No numeric cumulative release values. Only Beautifil II showed significant increase in enamel fluoride content (EDX: 1.51 → 1.67 At%)	GICs and RMGIC presented highest enamel hardness, stable levels of Ca, P and F. Beautiful II: intermediate hardness, partial protection, loss of Ca. Non-fluoridated composite: lowest hardness, highest demineralization.

## Data Availability

No new data were created or analyzed in this study. Data sharing is not applicable to this article.

## Supplementary Materials

The following supporting information can be downloaded at: https://www.mdpi.com/article/10.3390/ma18235350/s1, Table S1: Quality assessment of included studies.
