# Supplementary material for: Evaluation of Factors Affecting Fluoride Release from Dental Sealants: A Systematic Review
Source: Materials (Basel). 2025 Nov 27;18(23):5350. doi: 10.3390/ma18235350 (PMC12693251; doi:10.3390/ma18235350)
Supplement: Supplementary file 1 [file materials-18-05350-s001.zip › materials-3973772-supplementary.pdf]

This table presents the Joanna Briggs Institute (JBI) checklist for quasi-experimental studies, applied in the systematic review titled: **Evaluation of Factors Affecting Fluoride Release from Dental Sealants: A Systematic Review**

Maciej Dobrzyński [1,*], Sylwia Klimas [1], Agnieszka Kotela [2], Zuzanna Majchrzak [2], Julia Kensy [1], Marzena Laszczyńska [2], Witold Świenc [3], Natalia Grychowska-Gąsior [4], Magdalena Fast [5] and Jacek Matys [2,3,*]

[1] Department of Pediatric Dentistry and Preclinical Dentistry, Wroclaw Medical University, Krakowska 26, 50-425 Wroclaw, Poland; maciej.dobrzynski@umw.edu.pl (M.D.); sylwia.klimas@student.umw.edu.pl (S.K.); julia.kensy@student.umw.edu.pl (J.K.)
[2] Medical Center of Innovation, Wroclaw Medical University, Krakowska 26, 50-425 Wroclaw, Poland; kotela.agnieszka@gmail.com (A.K.); marzenalaszczynska@gmail.com; zuzanna.h.nawrocka@gmail.com (Z.M.)
[3] Dental Surgery Department, Wroclaw Medical University, Krakowska 26, 50-425 Wroclaw, Poland; jacek.matys@umw.edu.pl (J.M.); witold.swienc@umw.edu.pl (W.Ś.)
[4] Department of Dental Prosthetics, Wroclaw Medical University, Krakowska 26, 50-425 Wroclaw, Poland; natalia.grychowska@umw.edu.pl (N.G-G)
[5] Department of Drug Form Technology, Wroclaw Medical University, Borowska 211 A, 50-556 Wroclaw, Poland; magdalena.fast@umw.edu.pl (M.F.)

* Correspondence: maciej.dobrzynski@umw.edu.pl; jacek.matys@umw.edu.pl

Supplementary Table 1. Quality assessment of included studies.

Authors	1. Is It Clear in the Study What Is the 'Cause' and What Is the 'Effect'?	2. Were the Participants Included in Any Comparisons Similar?	3. Were the Participants Included in Any Comparisons Receiving Similar Treatment/Care, Other than the Exposure or Intervention of Interest?	4. Was There a Control Group?	5. Were There Multiple Measurements of the Outcome Both Pre- and Post-Intervention/Exposure?	6. Was Follow up Complete and If Not, Were Differences Between Groups in Terms of Their Follow up Adequately Described and Analyzed?	7. Were the Outcomes of Participants Included in Any Comparisons Measured in the Same Way?	8. Were Outcomes Measured in a Reliable Way?	9. Was Appropriate Statistical Analysis Used?
Thwe Zin [56]	Yes	Yes	Yes	Yes	Yes	Yes	Yes	Yes	Yes
Şişmanoğlu [57]	Yes	Yes	Yes	No	Yes	Yes	Yes	Yes	Yes
Fita [58]	Yes	Yes	Yes	No	Yes	Yes	Yes	Yes	Yes

Kantovitz [59]	Yes	Yes	Yes	No	Yes	Yes	Yes	Yes	Yes
Prapansilp [60]	Yes	Yes	Yes	Yes	Yes	Yes	Yes	Yes	Yes
Asvanund [61]	Yes	Yes	Yes	Yes	Yes	Yes	Yes	Yes	Yes
Dionysopoulos [62]	Yes	Yes	Yes	Yes	Yes	Yes	Yes	Yes	Yes
Poggio [63]	Yes	Yes	Yes	Yes	Yes	Yes	Yes	Yes	Yes
Ananda [64]	Yes	Yes	Yes	Yes	Yes	Yes	Yes	Yes	Yes
Fan [65]	Yes	Yes	Yes	Yes	Yes	Yes	Yes	Yes	Yes
Kaga [66]	Yes	Yes	Yes	Yes	Yes	Yes	Yes	Yes	Yes
Koga [67]	Yes	Yes	Yes	Yes	Yes	Yes	Yes	Yes	Yes
Kusgöz [68]	Yes	Yes	Yes	Yes	Yes	Yes	Yes	Yes	Yes
Kamala [69]	Yes	Yes	Yes	Yes	Yes	Yes	Yes	Yes	Yes
Lobo [70]	Yes	Yes	Yes	Yes	Yes	Yes	Yes	Yes	Yes
Leao [71]	Yes	Yes	Yes	Yes	Yes	Yes	Yes	Yes	Yes
